# The Effect of Aerobic Exercise in Neuroplasticity, Learning, and Cognition: A Systematic Review

**DOI:** 10.7759/cureus.54021

**Published:** 2024-02-11

**Authors:** Silvia Grimanesa Revelo Herrera, Jose E Leon-Rojas

**Affiliations:** 1 Psychology, Universidad Nacional de Chimborazo, Riobamba, ECU; 2 Neurological Surgery, Universidad de las Américas, Quito, ECU

**Keywords:** cycling, cortical excitability, neurogenesis and neuroplasticity, exercise training, aerobic exercise

## Abstract

This systematic review aims to examine the association between physical activity, neuroplasticity, and cognition. We analyzed an initial dataset consisting of 9935 articles retrieved from three scientific platforms (PubMed, Scopus, and the Virtual Health Library). Various screening filters were applied to refine the information against predefined eligibility criteria, resulting in the inclusion of a total of 17 articles that assessed the effect of aerobic exercise on neuroplasticity. The results suggested that aerobic exercise at various intensities, particularly at high intensity, can influence cortical excitability and result in cognitive improvement; also, exercise was associated with direct cortical and structural changes. Exercise has shown efficacy in individuals of diverse age groups, as well as in people with and without brain disease.

## Introduction and background

Synaptic plasticity refers to the inherent capacity of the nervous system to modulate the strength of neuronal interactions and regeneration [1]. It is widely acknowledged that engaging in regular exercise yields advantageous effects on cognitive and brain functions [1-3]. Furthermore, apart from its conventional recommendation for enhancing cardiopulmonary and musculoskeletal well-being, it has been firmly established that physical activity also exerts notable influences on brain functionality [4]. For instance, research has demonstrated that a solitary exercise session can alter synaptic communication, resulting in enhanced executive functions such as planning, task switching, response inhibition, and working memory [1,4-7]. The existing body of evidence primarily consists of studies involving moderate-intensity, multi-session exercise across various age groups [3]. However, recent findings indicate that engaging in higher-intensity exercise enhances memory in children, suggesting that exercise has cognitive benefits, including improved memory function, that persist throughout the lifespan [8]. 

The molecular aspect of the neurobiology of neurogenesis is also influenced by aerobic exercise as it creates a cellular environment that facilitates the enhancement of neuronal development, survival, and activity in the hippocampus; this process entails the enhancement of the synthesis and secretion of brain-derived neurotrophic factor (BDNF), a crucial neurotransmitter involved in the facilitation of cognitive functions, along with an augmentation in receptor density [3,4,7,9-11]. Changes of this factor have been previously reported. For example, studies have shown that women tend to have a more pronounced elevation of BDNF during aerobic exercise, as compared to males [6,7,9]. Moreover, it has been noted that individuals diagnosed with neurodegenerative diseases and a range of psychiatric conditions, including depression, post-traumatic stress disorder, schizophrenia, obsessive-compulsive disorder, autism, bipolar disorder, addiction, and depressive disorder, exhibit diminished levels of BDNF [12]. 

Changes in BDNF appear to not only occur in disease but also as a response to therapy with various drugs such as antidepressant, antipsychotic, and euthymic medicines that increase its levels [12]. Additionally, non-pharmacological interventions, including electroconvulsive treatment and transcranial magnetic stimulation, have also been explored for their effects on BDNF levels and are widely recognized as effective interventions [12,13]. As mentioned before, aerobic exercise leads to an elevation of BDNF levels, not only in the brain but also in the bloodstream, inducing an antidepressant effect, both when used independently and in conjunction with pharmacological and non-pharmacological treatments [10,14]. Certainly, aerobic activities have been shown to have an effect on mood via several mechanisms that include enhancing self-efficacy, motivation, and energy levels [10,12-14]. Additionally, aerobic workouts have been shown to improve psychosocial functioning and induce antidepressant effects through promoting beneficial neurobiological changes [10,12-18]. 

Therefore, the primary aim of our systematic review is to synthesize existing scientific literature that looks into the interplay between neuroplasticity and exercise. Furthermore, an assessment will be conducted on the quality and potential biases of the included studies.

## Review

Methodology

Our systematic review followed the requirements set out by the Preferred Reporting Items for Systematic Reviews and Meta-Analysis (PRISMA) [15]. 

Eligibility Criteria 

The selection criteria for the articles were limited to those that provided a comprehensive examination of the impact of exercise on neuroplasticity and its implications. Articles that incorporated randomized clinical trials and diagnostic studies were also taken into account, encompassing any study that investigated the influence of exercise on neuroplasticity in humans. These studies considered various methodologies, including imaging studies, clinical investigations, analyses of human cell samples, and examinations of human tissues. The exclusion criteria for the papers included in the analysis were case reports, literature reviews, systematic reviews, meta-analyses, letters to the editor, and conference abstracts. Articles published only in Spanish and English were considered for this study.

Information Sources 

The search method included medical subheadings (MeSH) and text phrases pertaining to neuroplasticity, physical activity, and exercise. The databases included in this research encompassed Medline (PubMed), Scopus, and Virtual Health Library (BVS), spanning from inception to January 27, 2023.

Search Strategy 

The search criteria used in the databases did not include any limitations or constraints. A comprehensive and methodical exploration was undertaken in Scopus (commencing on January 27, 2023), MEDLINE (commencing on January 27, 2023), and BVS (commencing on January 27, 2023) using the following key terms: “neuroplasticity”, “neuronal plasticity”, and “exercise”. We performed an independent and blinded evaluation of the publications. In instances where there were discrepancies among the selected articles, we engaged in discussions until a consensus was achieved. 

Data Management

The articles obtained from the databases were loaded into Ryyan, a web-based program [16], in order to reduce data input mistakes and eliminate bias during the deduplication and decision process. 

Selection Process 

We used the aforementioned inclusion criteria to determine the selection of all titles and abstracts. Subsequently, the literature that satisfied the predetermined inclusion criteria, regardless of the level of certainty in the findings, received a comprehensive evaluation of the whole text to determine its suitability. All decision-making was done in a blinded manner between the two reviewers.

Data Items

The data obtained from the chosen publications was gathered and systematically arranged in a Microsoft Excel spreadsheet. The citation details of the paper were provided, including the author's name, year of publication, country of origin, and research design. Data was collected on the sample size, gender distribution, age demographics, pre-training and post-training evaluations, exercise type, as well as the specific instrument or test used for assessing and monitoring the outcomes. The synthesis of the data was conducted using proportions due to the substantial variability seen among individuals and the use of several tests to assess the impact of exercise on neuroplasticity. 

Bias Assessment 

We used the National Heart, Lung, and Blood Institute (NHLBI) study quality assessment tools in order to evaluate the likelihood of bias in the included studies. The degree of bias was classified as low, moderate or high based on the proportion of “yes” replies to the questions posed by each design-specific questionnaire. A low risk of bias was determined when at least 80% of the questions were answered in a positive manner; a moderate risk of bias was attributed when the proportion of positive responses fell between the range of 50% to 79%; and, conversely, a high risk of bias was awarded when fewer than 50% of the questions were answered in a positive manner. 

Results

**Figure 1:**
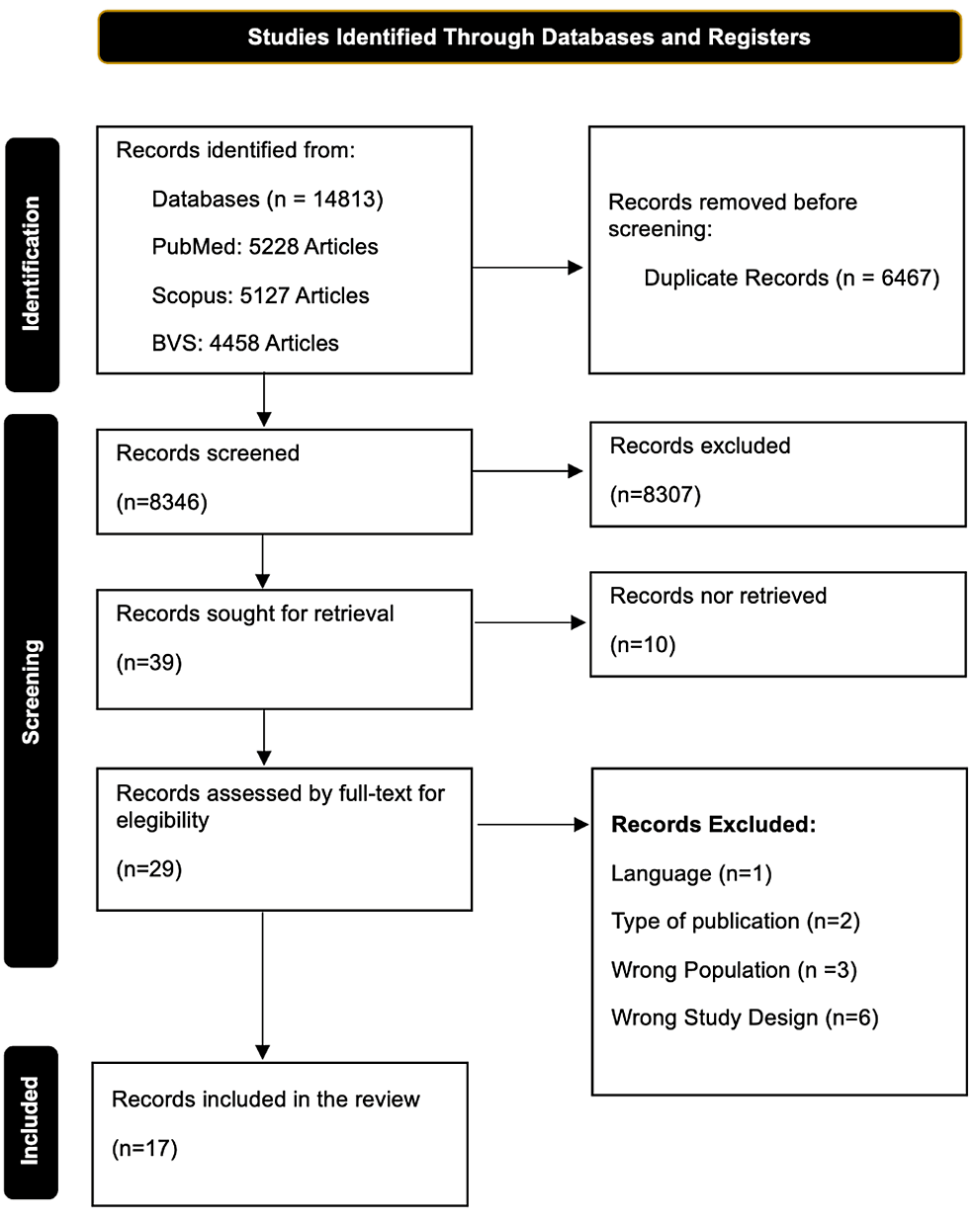
PRISMA 2020 flowchart showcasing the article selection process BVS: Virtual Health Library; PRISMA: Preferred Reporting Items for Systematic Reviews and Meta-Analyses

The current investigation included a thorough examination and evaluation of scholarly publications sourced from three distinct scientific platforms. This process yielded a total of 9935 articles, which were further assessed for inclusion by two impartial reviewers who were unaware of each other's evaluations. The procedure of selecting articles and the outcomes of this approach is shown in Figure 1. In all, 17 publications were chosen for the purpose of this review [1-14,17,19,20], including a sample size of 755 individuals whose ages ranged from nine to 83 years. The bias evaluation conducted for each individual study can be seen in Table 1. In total, 56% of the studies exhibited a moderate risk of bias, while 44% showed low risk of bias. 

**Table 1:** Bias assessment of each study

AUTHOR (YEAR)	STUDY DESIGN	RISK OF BIAS
Rogge AK (2018)[2]	Randomized Controlled Trial	Low
Reycraft JT (2020)[17]	Nonrandomized, Pre-Post Intervention	Moderate
Gomes-Osman J (2017)[9]	Nonrandomized, Pre-Post Intervention	Low
Mang CS (2026)[4]	Nonrandomized, Pre-Post Intervention	Moderate
Pareja-Galeano H (2013)[14]	Cross-Sectional	Moderate
Ross RE (2019)[12]	Randomized Controlled Trial	Moderate
Hiroyuki Shimada (2017)[13]	Randomized Controlled Trial	Moderate
Chapman SB (2013)[10]	Randomized Controlled Trial	Low
Kubica J (2019)[11]	Randomized Controlled Trial	Moderate
Best J (2015)[19]	Randomized Controlled Trial	Low
Filipas (2020)[5]	Randomized Controlled Trial	Moderate
Morris TP (2020)[3]	Nonrandomized, Pre-Post Intervention	Low
Schneider S (2009)[6]	Nonrandomized, Pre-Post Intervention	Low
El-Sayes (2019)[20]	Nonrandomized, Pre-Post Intervention	Low
Andrews SC (2020)[1]	Nonrandomized, Pre-Post Intervention	Moderate
Perini R (2016)[7]	Randomized Controlled Trial	Moderate
Loprinzi PD (2019)[8]	Randomized Controlled Trial	Moderate

Exercise, as mentioned before, appears to have a significant effect on both cortical activation of key areas related to normal cognitive function as well as in the release of BNDF. In our study, six articles assess the changes of BDNF secondary to exercise activity, including a total of 80 participants with ages ranging from nine to 67 years [6,9,11,12,14,17]. In the pediatric groups, Schneider et al. conducted a study involving 11 children between the ages of nine and 11, which revealed a significant increase in alpha activity, assessed by employing low resolution electromagnetic tomography (LORETA), when performing exercise within the superior parietal lobe and precuneus, encompassing substantial portions of Brodman's area 7 (p<0.05) [6]. In another study focusing on adolescents, those who were trained cyclists (with an average of five training sessions and 19 hours per week) exhibited a noteworthy elevation in plasma levels of BDNF in comparison to their sedentary counterparts (1815.3 ± 948.6 pg/mL vs. 570.7 ± 516.8 pg/mL) [14]. Similarly, Reycraft et al. reported a significant increase in plasma BDNF levels in adolescents and young adults performing moderate-intensity continuous training, vigorous-intensity continuous training, and sprint interval training; interestingly enough, BDNF levels increased more rapidly in the sprint interval training group and these levels were significantly higher than in any of the other groups (p=0.002) [17].

In young and middle-aged adults, a similar benefit is noted immediately after exercise with regards to an increase in BDNF [9,12]. Gomes-Osman et al. included 14 participants, 27 years on average, who were exposed to 16 aerobic exercise sessions (four times per week for four weeks) on a treadmill. A saliva sample was collected for BDNF analysis of Val66Val and Val66Met mutations of the BDNF gene; after the exercise intervention there was better response inhibition measured in the Stroop Test (p=0.05) in all groups and the Val66Val group exhibited increased performance when compared with the Met carriers [9]. On the other hand, a study that included 26 participants, 27.2 year on average, 13 with depression and 13 controls, subjected to either 15 minutes of low-intensity cycling (at 35% of maximum heart rate), high-intensity cycling (at 70% of maximum heart rate), or sitting without cycling showed that postexercise BDNF significantly increased in both groups, particularly after high-intensity exercise (6.91 ng/mL [95%CI = 5.54 - 8.29]) when compared to low-intensity (3.24 ng/mL [95%CI = 2.04 - 4.44]), and sitting (0.94 ng/mL [95%CI = −0.50 - 2.38]) (p<0.01). Furthermore, linear regression analysis showed that BDNF increased 0.71 ng/mL for every 10 BPM increase in heart rate from baseline (p<0.001) [12]; showcasing the importance of exercise intensity for biomolecular changes in the brain after exercise. Finally, on the other extreme of age, an article published by Kubica et al. looked into the association between physical exercise (classic balance training) and neuroplasticity in a sample of 74 elder individuals, with a mean age of 65.34 ± 3.79 years [11]. The results of this study showed that in individuals who underwent training in comparison with controls, there was a significant increase of activity in the supplementary motor area (p<0.05), the left and right supramarginal gyrus/posterior insula (left: p<0.05; right: p<0.05), the middle occipital gyrus (laterally)/area V5 (left: p<0.05; right: p<0.05), and the cerebellum-inferior semilunar lobe/tonsil (p<0.05) [11]. Additionally, following the completion of the training regimen, serum concentrations of BDNF exhibited a significant rise in the intervention group when compared to controls (p<0.05) [11].

On the other hand, exercise also seems to have a direct effect on the cerebral cortex and its neuroplasticity, particularly on excitability. A total of 115 participants, with average ages ranging from 21 to 35.10 years, were included in four scientific articles that subjected them to varying degrees of aerobic exercise (particularly cycling) and measured their cortical excitability [1,3,4,20]. In one study, 14 participants were subjected to light aerobic exercise consisting of 30 minutes of static cycling for two sessions separated by seven days. A multitasking neurocognitive test was applied and showed a significant improvement after exercise and a decrease in performance during rest (p=0.003); additionally, despite the short aerobic session, cortical excitability also showed a significant increase [3]. Similarly, El-Sayes et al. subjected 34 participants to 20 minutes of continuous cycling of moderate intensity (65-70% of the maximum heart rate) at a steady rate between 70-90 revolutions per minute. During exercise, cortical and muscle excitability were measured and an increase excitability in the corticospinal tract and neuroplasticity in both men and women was shown (p=0.02); there were no differences in cortical excitability between males and females [20]. In another study, 47 participants performed high-intensity cycling (90% of the maximum heart rate) until failure by increasing intensity every two minutes. Cortical excitability was measured and it showed that short-term aerobic exercise induces excitability in the cerebellar circuitry and increases plasticity which might lead to improvement in motor skills [4]. Finally, Andrews et al. subjected 20 participants to static 20-minute cycling sessions with high-intensity intervals or moderate continuous cycling; they also received transcranial magnetic stimulation (TMS) after the exercise sessions. Only the participants subjected to the high-intensity interval training showed a significant effect on cortical excitability (p=0.003) when compared to the moderate-intensity group, despite this last group also receiving TMS [1]. As shown, exercise, particularly aerobic exercise, at higher intensities is associated with increased cortical plasticity and measurable cortical excitability in both cerebral and cerebellar cortices.

Apart from the biochemical (BDNF) and stimulatory effects (cortical excitability) of exercise, we also found studies reporting structural changes in the brain. For instance, a study involving 37 participants subjected to a regime of exercise twice a week for 12 weeks and balance training showed that cortical thickness increased in the superior temporal cortex, visual association cortex, posterior cingulate cortex, and the precentral gyrus; additionally, putaminal atrophy was reduced in the training group, significantly improving balance [2]. Another study in elder adults (average age of 78 years) that measured changes in brain metabolism with positron emission tomography (PET) with 18-fluoro-desoxyglucose (18FDG) showed that the exercise group had a significant increase in glucose metabolism in the premotor and supplementary motor areas, the somatosensory association cortices and the primary visual cortex after bimonthly aerobic exercise and strength training for three months [13]. Additionally, in comparison with the control group, the intervention group showed a significant increase in glucose uptake in the left posterior entorhinal cortex (p=0.001), the left superior temporal gyrus (p<0.05), and in the right superior temporal pole area (p<0.01); which can aid in preventing future disabilities in elder adults [13].

Finally, when assessing the effect of exercise on cognition, we found five articles encompassing a total of 376 participants ranging from 18 to 75 years of age [5,7,8,10,19]. The largest study involved 155 elder adults (65-75 years old) who were placed on a training regime, consisting of 60-minute sessions of resistance training progressing in intensity as well as stretching, balance, and range of motion exercises for 52 weeks with a follow-up immediately after the regime and in two years [19]. In this study, resistance training, once per week, showed a positive impact in executive functions and memory at the end of the 52-week period and this effect was maintained after two years of follow-up (p=0.005); additionally, white matter atrophy was also reduced during the follow-up period [19]. Another study in older adults included 37 participants (57-75 years old), divided into an exercise and a control group; the exercise group underwent aerobic training (cycling and treadmill) for three one-hour sessions every week for a total of 12 weeks. All participants received a cognitive, cardiovascular and cerebral blood flow assessment at the beginning, middle and end of the 12-week regimen [10]. Cerebral blood flow and cognition significantly improved in the exercise group at the middle and end of the follow-up (p=0.003 and p=0.03, respectively) compared with the control group [10]. The other three articles assessing cognition involved younger adults ranging from 18 to 35 years of age, all subjected to aerobic exercise (cycling or treadmill). The first study included 20 participants subjected to four weeks of resistance training (cycling) and showed that participants had a higher tolerance for mental fatigue after exercise, particularly resistance training that increased progressively in intensity until failure [5]. Apparently, experiencing physical exertion and fatigue also trains the brain to progressively tolerate mental exertion and mental fatigue. The second study involved 84 male participants cycling at 60 revolutions per minute for 30 minutes and divided into two groups, one cycling at a moderate intensity (70% of the maximum heart rate) and a control group cycling at a very low rate; both groups were subjected to learning tasks. This study showed that the learning rate in both groups was different (p=0.037) favoring the moderate-intensity exercise group even after only one exercise session [7]. The third study included 80 participants divided into four groups: (1), exercise and learning; (2), only learning; (3), only exercise; and (4), neither (control). This study showed a strong effect of learning techniques, such as the 3R technique, in improving learning and also showed a synergistic effect of acute exercise in learning (stronger benefit in group 1) [8]. However, is important to note that when exercise was assessed without the learning techniques, the benefit was significantly reduced.

Discussion

This review showcases the potential advantageous effects of exercise on brain health; as mentioned before, even a few sessions of exercise (with better effects the higher the intensity) have a significantly positive effect on the biochemical, synaptic, structural, functional, and cognitive spheres by having a direct influence in BDNF, cortical excitability, cortical thickness, glucose metabolism, and executive functioning (learning), respectively.

BDNF is an essential protein that has a major function in facilitating the growth, maturation, and sustenance of neurons in the brain [21]. Increased levels of BDNF have been associated with a wide range of beneficial impacts on brain health such as improved neuroplasticity, which is the brain's ability to adapt and change [21]. Additionally, BDNF facilitates synaptic development, the formation of connections between neurons, and enhances neuronal survival; the enhanced plasticity is essential for acquiring knowledge, retaining information, and adjusting to novel encounters [21,22]. Moreover, elevated levels of BDNF have been associated with enhanced cognitive performance. BDNF facilitates the creation of fresh memories and aids in the solidification of knowledge [21,23]. Research has shown that people with elevated levels of BDNF exhibit superior performance in activities associated with memory and learning [23]. This neurotrophic factor also has a vital purpose in safeguarding neurons from harm and enhancing their lifespan, which is necessary for preserving cognitive function as we become older. As shown by our review, BDNF increases significantly after exercise, particularly high-intensity regimes irrespective of age [6,9,11,12,14,17], and as exemplified above, such increase has multiple benefits ranging from neuroplasticity to synaptic maintenance, neuronal survival, and neuroprotection. Certainly, engaging in physical exercise is a very effective natural method for boosting levels of BDNF. The evidence provided here shows that engaging in consistent physical exercise amplifies the synthesis of BDNF, which offers a logical rationale for the beneficial effects of physical activity on brain well-being. The production of BDNF during exercise is linked not just to enhanced cognitive performance but also to the prevention of cognitive loss associated with aging.

Cortical excitability, which refers to the extent of neuronal responsiveness to stimuli in the cerebral cortex, plays a crucial role in defining the overall health and functioning of the brain. Proper cognitive processing, sensory perception, and motor control rely on maintaining a crucial equilibrium between excitation and inhibition in the cortex [24]. Efficient neural communication relies on optimal cortical excitability, which facilitates the establishment and strengthening of neural networks involved in different cognitive processes [24]. Alterations in cortical excitability have been linked to many neurological and psychiatric illnesses, underscoring its importance in preserving brain well-being [25-27]. Furthermore, cortical excitability is crucial in influencing neuronal plasticity; the acquisition and retention of knowledge greatly depend on synaptic plasticity, a phenomenon in which the excitability of the cortex affects the potency and efficiency of synaptic connections [28,29]. Regulating cortical excitability is crucial for achieving optimum cognitive function and facilitating memory formation [28,29]. Studies indicate that engaging in activities that maintain a harmonious level of cortical excitability, such as consistent physical activity, sufficient sleep, and effective stress management, have a significant role in fostering optimal brain function and well-being [24,28,29]. Certainly, as shown in our review, exercise has a direct effect on cortical excitability in both the cerebral and cerebellar cortices, especially high-intensity exercise [1,3,4,20]. The findings in our review match those found in basic science and animal studies showcasing that neuronal excitability and stimulation foster stronger connections and therefore a longer lasting effect.

Finally, regular physical activity has been identified as a powerful regulator of cognitive performance, particularly in relation to memory in the literature. Participating in physical exercise, whether it is aerobic or strength training, has been linked to enhancements in several parts of memory, especially in the hippocampus, a crucial brain area for learning and memory functions [10,19]. As explained before, physical activity stimulates the secretion of neurotrophic factors, such as BDNF, which facilitates the development and preservation of nerve cells, supports the adaptability of synapses, and improves the creation of fresh memories [6,9,11,12,14,17]. Research has shown that engaging in aerobic activities, such as running or swimming, may enhance the size of the hippocampus and enhance spatial memory [30]. Moreover, resistance training has shown beneficial impacts on verbal memory [30]. The cognitive advantages of these methods are achieved by improved cerebral blood flow, heightened neurotransmitter synthesis, and the establishment of a neurochemical milieu that supports neuroplasticity [10]. Exercise-induced alterations not only enhance immediate cognitive functioning but also enhance the brain's ability to withstand challenges, thereby lessening the impact of age-related cognitive decline and decreasing the likelihood of neurodegenerative disorders [10,19]. The growing body of data presented here strongly supports the incorporation of regular exercise into one's lifestyle as a proactive approach to promote cognitive health and enhance memory performance [5,7,8,10,19]. As shown in our review, aerobic exercise results in immediate and long-term results for individuals of any age and seems to be particularly beneficial in memory, cognitive flexibility, executive functioning and mental fatigue.

To summarize, our systematic analysis uncovers a convincing body of data affirming the beneficial influence of exercise on several facets of brain health across individuals of various age groups. The reviewed research consistently shows that exercise has an impact on brain function at several levels, including biochemical, stimulatory, and structural aspects. Exercise has a crucial role in stimulating the production of BDNF, which is essential for improving cognitive performance. Research repeatedly demonstrates that various types and amounts of physical activity led to increased levels of BDNF, a protein linked with enhanced cognitive function, across diverse age groups ranging from toddlers to elderly persons. It is worth mentioning that workouts with greater intensity, such as sprint interval training and high-intensity cycling, have a faster and more noticeable impact on BDNF levels. This highlights the significance of exercise intensity in impacting biochemical alterations in the brain.

Additionally, engaging in physical activity, especially aerobic exercises, seems to regulate the level of cortical excitability, so enhancing the adaptability of both the cerebral and cerebellar cortices. The results indicate that exercise-induced neuroplasticity is vital for enhancing brain function and perhaps enhancing motor abilities. These also can result in structural modifications in the brain, including an increase in the thickness of the cortex, changes in brain metabolism, and a decrease in the degeneration of white matter in different areas. These structural modifications are linked to enhanced equilibrium, cognitive ability, and the avoidance of impairments, especially among the elderly.

Ultimately, the influence of physical activity on cognitive function is seen across individuals of all age brackets. Resistance training and aerobic workouts have been shown to have beneficial impacts on executive functioning, memory, and the ability to tolerate mental fatigue. The significant impact of combining exercise and learning strategies highlights the possibility for joint therapies to improve cognitive results. Therefore, further research should focus on elucidating the precise processes that underlie these effects and enhancing exercise recommendations to optimize cognitive advantages.

## Conclusions

Studies have shown over and over that exercise changes the way the brain works on biochemical, stimulatory, and structural levels. It plays a key role in increasing the production of BDNF, which improves brain functionality. Notably, different types and intensities of physical activity lead to different benefits, emphasizing the importance of exercise intensity in influencing biochemical and structural changes in the brain. Aerobic exercises, in particular, regulate cortical excitability, contributing to enhanced adaptability in cerebral and cerebellar cortices. Finally, exercise, even when performed in fewer weekly sessions, has a positive cognitive impact on individuals of all ages.

## References

[1] Andrews SC, Curtin D, Hawi Z, Wongtrakun J, Stout JC, Coxon JP (2020). Intensity matters: high-intensity interval exercise enhances motor cortex plasticity more than moderate exercise. Cereb Cortex.

[2] Rogge AK, Röder B, Zech A, Hötting K (2018). Exercise-induced neuroplasticity: balance training increases cortical thickness in visual and vestibular cortical regions. Neuroimage.

[3] Morris TP, Fried PJ, Macone J (2020). Light aerobic exercise modulates executive function and cortical excitability. Eur J Neurosci.

[4] Mang CS, Brown KE, Neva JL, Snow NJ, Campbell KL, Boyd LA (2016). Promoting motor cortical plasticity with acute aerobic exercise: a role for cerebellar circuits. Neural Plast.

[5] Filipas L, Martin K, Northey JM, La Torre A, Keegan R, Rattray B (2020). A 4-week endurance training program improves tolerance to mental exertion in untrained individuals. J Sci Med Sport.

[6] Schneider S, Vogt T, Frysch J, Guardiera P, Strüder HK (2009). School sport--a neurophysiological approach. Neurosci Lett.

[7] Perini R, Bortoletto M, Capogrosso M, Fertonani A, Miniussi C (2016). Acute effects of aerobic exercise promote learning. Sci Rep.

[8] Loprinzi PD, Harris F, McRaney K (2019). Effects of acute exercise and learning strategy implementation on memory function. Medicina (Kaunas).

[9] Gomes-Osman J, Cabral DF, Hinchman C, Jannati A, Morris TP, Pascual-Leone A (2017). The effects of exercise on cognitive function and brain plasticity - a feasibility trial. Restor Neurol Neurosci.

[10] Chapman SB, Aslan S, Spence JS, Defina LF, Keebler MW, Didehbani N, Lu H (2013). Shorter term aerobic exercise improves brain, cognition, and cardiovascular fitness in aging. Front Aging Neurosci.

[11] Kubica J, Szymura J, Domagalik A (2019). Systematic balance exercises influence cortical activation and serum BDNF levels in older adults. J Clin Med.

[12] Ross RE, Saladin ME, George MS, Gregory CM (2019). High-intensity aerobic exercise acutely increases brain-derived neurotrophic factor. Med Sci Sports Exerc.

[13] Shimada H, Ishii K, Makizako H, Ishiwata K, Oda K, Suzukawa M (2017). Effects of exercise on brain activity during walking in older adults: a randomized controlled trial. J Neuroeng Rehabil.

[14] Pareja-Galeano H, Brioche T, Sanchis-Gomar F (2013). Impact of exercise training on neuroplasticity-related growth factors in adolescents. J Musculoskelet Neuronal Interact.

[15] Page MJ, Moher D, Bossuyt PM (2021). PRISMA 2020 explanation and elaboration: updated guidance and exemplars for reporting systematic reviews. BMJ.

[16] Ouzzani M, Hammady H, Fedorowicz Z, Elmagarmid A (2016). Rayyan-a web and mobile app for systematic reviews. Syst Rev.

[17] Reycraft JT, Islam H, Townsend LK, Hayward GC, Hazell TJ, Macpherson RE (2020). Exercise intensity and recovery on circulating brain-derived neurotrophic factor. Med Sci Sports Exerc.

[18] Roeh A, Bunse T, Lembeck M (2020). Running effects on cognition and plasticity (ReCaP): study protocol of a longitudinal examination of multimodal adaptations of marathon running. Res Sports Med.

[19] Best JR, Chiu BK, Liang Hsu C, Nagamatsu LS, Liu-Ambrose T (2015). Long-term effects of resistance exercise training on cognition and brain volume in older women: results from a randomized controlled trial. J Int Neuropsychol Soc.

[20] El-Sayes J, Turco CV, Skelly LE, Nicolini C, Fahnestock M, Gibala MJ, Nelson AJ (2019). The effects of biological sex and ovarian hormones on exercise-induced neuroplasticity. Neuroscience.

[21] Rothman SM, Mattson MP (2013). Activity-dependent, stress-responsive BDNF signaling and the quest for optimal brain health and resilience throughout the lifespan. Neuroscience.

[22] Kowiański P, Lietzau G, Czuba E, Waśkow M, Steliga A, Moryś J (2018). BDNF: a key factor with multipotent impact on brain signaling and synaptic plasticity. Cell Mol Neurobiol.

[23] Bekinschtein P, Cammarota M, Medina JH (2014). BDNF and memory processing. Neuropharmacology.

[24] Cespón J, Pellicciari MC, Casula EP, Miniussi C (2022). Age-related changes in cortical excitability linked to decreased attentional and inhibitory control. Neuroscience.

[25] Wang Y, Ramandi D, Sepers MD, Mackay JP, Raymond LA (2023). Age- and region-dependent cortical excitability in the zQ175 Huntington disease mouse model. Hum Mol Genet.

[26] King R, Kirton A, Zewdie E, Seeger TA, Ciechanski P, Barlow KM (2019). Longitudinal assessment of cortical excitability in children and adolescents with mild traumatic brain injury and persistent post-concussive symptoms. Front Neurol.

[27] Shin SS, Krishnan V, Stokes W (2018). Transcranial magnetic stimulation and environmental enrichment enhances cortical excitability and functional outcomes after traumatic brain injury. Brain Stimul.

[28] Centeno C, Medeiros D, Beck MM, Lugassy L, Gonzalez DF, Nepveu JF, Roig M (2018). The effects of aging on cortico-spinal excitability and motor memory consolidation. Neurobiol Aging.

[29] Reinhart RM, McClenahan LJ, Woodman GF (2016). Attention's accelerator. Psychol Sci.

[30] Erickson KI, Voss MW, Prakash RS (2011). Exercise training increases size of hippocampus and improves memory. Proc Natl Acad Sci U S A.

